# MiR-146a-5p suppresses activation and proliferation of hepatic stellate cells in nonalcoholic fibrosing steatohepatitis through directly targeting Wnt1 and Wnt5a

**DOI:** 10.1038/srep16163

**Published:** 2015-11-05

**Authors:** Jinghua Du, Xuemin Niu, Yang Wang, Lingbo Kong, Rongqi Wang, Yuguo Zhang, Suxian Zhao, Yuemin Nan

**Affiliations:** 1 Department of Traditional and Western Medical Hepatology, Third Hospital of Hebei Medical University, Shijiazhuang, China

## Abstract

Nonalcoholic fibrosing steatohepatitis is a uniform process throughout nonalcoholic fatty liver disease (NAFLD). MicroRNAs (miRNAs) have been suggested to modulate cellular processes in liver diseases. However, the functional role of miRNAs in nonalcoholic fibrosing steatohepatitis is largely unclear. In this study, we systematically analyzed the hepatic miRNAs by microarray analysis in nonalcoholic fibrosing steatohepatitis in C57BL/6J mice induced by methionine-choline deficient (MCD) diet. We identified 19 up-regulated and 18 down-regulated miRNAs in liver with fibrosis. Among these dysregulated miRNAs, miR-146a-5p was the most significant down-regulated miRNA. Luciferase activity assay confirmed that Wnt1 and Wnt5a were both the target genes of miR-146a-5p. Hepatic miR-146a-5p was down-regulated in fibrosing steatohepatitis, but its target genes Wnt1 and Wnt5a and their consequent effectors α-SMA and Col-1 were significantly up-regulated. In addition, miR-146a-5p was downregulated, whilst Wnt1 and Wnt5a were up-regulated in the activated primary hepatic stellate cells (HSCs) compared to the quiescent primary HSCs. Overexpression of miR-146a-5p in HSCs inhibited HSC activation and proliferation, which concomitant with the decreased expressions of Wnt1, Wnt5a, α-SMA and Col-1. In conclusion, miR-146a-5p suppresses activation and proliferation of HSCs in the progress of nonalcoholic fibrosing steatohepatitis through targeting Wnt1 and Wnt5a and consequent effectors α-SMA and Col-1.

Nonalcoholic steatohepatitis (NASH) is part of the spectrum of nonalcoholic fatty liver disease (NAFLD), characterized by steatosis, lobular inflammation and progressive pericellular fibrosis^1^. With prolonged liver damage, steatohepatitis may progress to liver fibrosis featured with the excessive accumulation of extracellular matrix (ECM). Hepatic stellate cells (HSCs) play a central role in the pathogenesis of liver fibrosis^2^. Quiescent HSCs can be activated in response to chronic steatohepatitis^3^. Activated HSCs stimulate the collagen production and ECM accumulation, which results in the occurrence of liver fibrosis^4^. Despite fundamental advances in understanding the pathophysiology of nonalcoholic fibrosing steatohepatitis, the mechanisms of fibrogenesis in the presence of steatohepatitis remain elusive.

MicroRNAs (miRNAs) are a class of short non-coding RNAs, about 19–22 nucleotides in length, that can bind to the 3′-untranlated regions (3′UTR) in target mRNA molecules, causing translation repression or the cleavage of the target mRNAs^5^. miRNAs can recognize hundreds of targets genes with the incomplete complementary sequences and over one third of human genes appear to be conserved miRNAs targets^6^. Many studies demonstrated that miRNAs play essential roles in a variety of cellular processes such as metabolism, immune function, cell proliferation, and apoptosis^7^,^8^,^9^. Aberrant expression of miRNAs is associated with a variety of liver diseases, including viral hepatitis, autoimmune liver disease and liver cancer^10^,^11^. Recent studies showed that miRNAs could regulate the activation of HSCs and fibrogenesis^12^,^13^,^14^. These may particularly contribute to the pathogenesis of nonalcoholic fibrosing steatohepatitis. However, the functional significance of miRNAs in the fibrogenesis process remains unclear. Identification of abnormally expressed miRNAs in the important pathologic state of NAFLD is helpful to further understand the molecular mechanism of nonalcoholic fibrosing steatohepatitis. In this study, we evaluated the differentially expressed miRNAs in nonalcoholic fibrosing steatohepatitis induced in mice fed with methionine-choline deficient (MCD) diet^15^,^16^,^17^ and demonstrated for the first time that miR-146a-5p suppresses activation and proliferation of HSCs in the pathogenesis of nonalcoholic fibrosing steatohepatitis through targeting Wnt1 and Wnt5a and their consequent effectors alpha-smooth muscle actin (α-SMA) and type I collagen (Col-1).

## Results

### Differential expression of hepatic miRNAs in mice with fibrosing steatohepatitis

**Figure 1:**
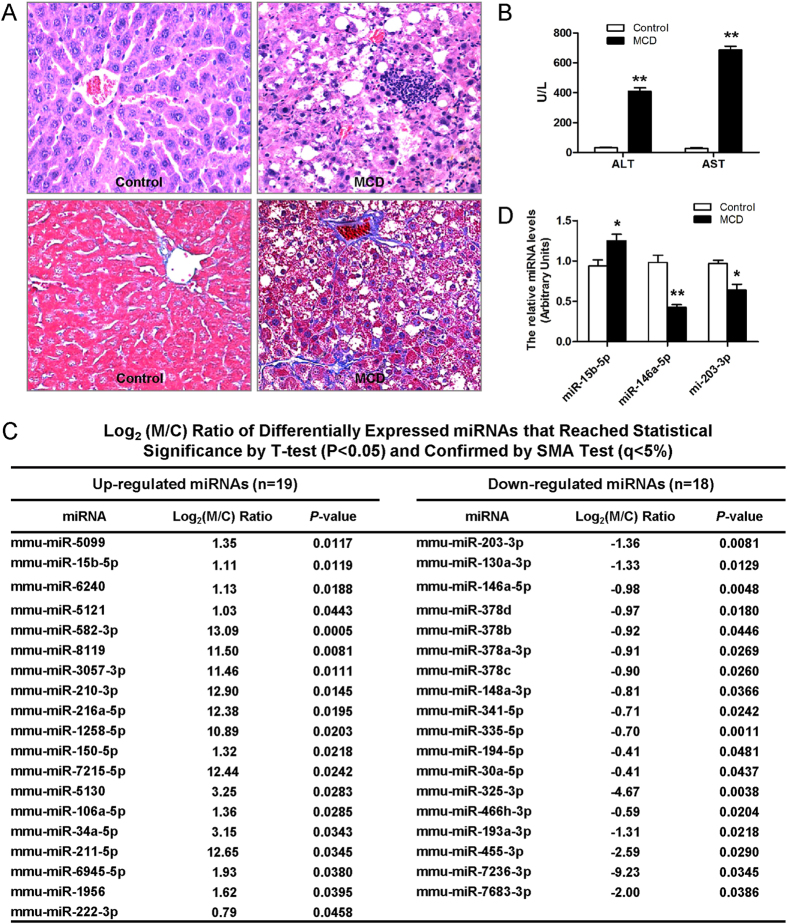
Histopathological changes of liver sections, miRNA microarray data and validation of differentially expressed miRNAs in mice. (**A**) Histopathological changes of liver sections in mice fed MCD diet and control. Hematoxylin and eosin stained (up) and Masson trichromatism stained (down). (**B**) Effect of MCD diet on serum ALT and AST levels. (**C**) Differentially regulated miRNAs as identified by miRNA microarray. (**D**) Validation of microarray data using real-time RT-PCR. Triplicate assays were done for each RNA sample and the relative amount of each miRNA was normalized to U6 snRNA. Values are mean ± SD, **P < 0.01 compared with control.

As shown in Fig. 1, the liver sections from mice fed an MCD diet exhibited disordered lobule structure, macrosteatosis in Zone 3, spot or focal hepatocyte necrosis, inflammatory infiltration and perisinusoidal fibrosis (Fig. 1A), which companied with significantly higher serum ALT and AST levels (*P* < 0.01) compared to the control diet-fed mice (Fig. 1B). To investigate the potential involvement of miRNAs in nutritional fibrosing steatohepatitis, we performed the miRNAs microarray analysis to assess the miRNAs expression profiles in the livers of mice with fibrosing steatohepatitis and those with normal histology. There were 37 dysregulated hepatic miRNAs in fibrosing steatohepatitis compared to the normal liver, including 19 up-regulated and 18 down-regulated miRNAs (>2-fold changes) (Fig. 1C).

### Microarray-based gene ontology analysis and pathway analysis for differentially expressed miRNAs

**Figure 2:**
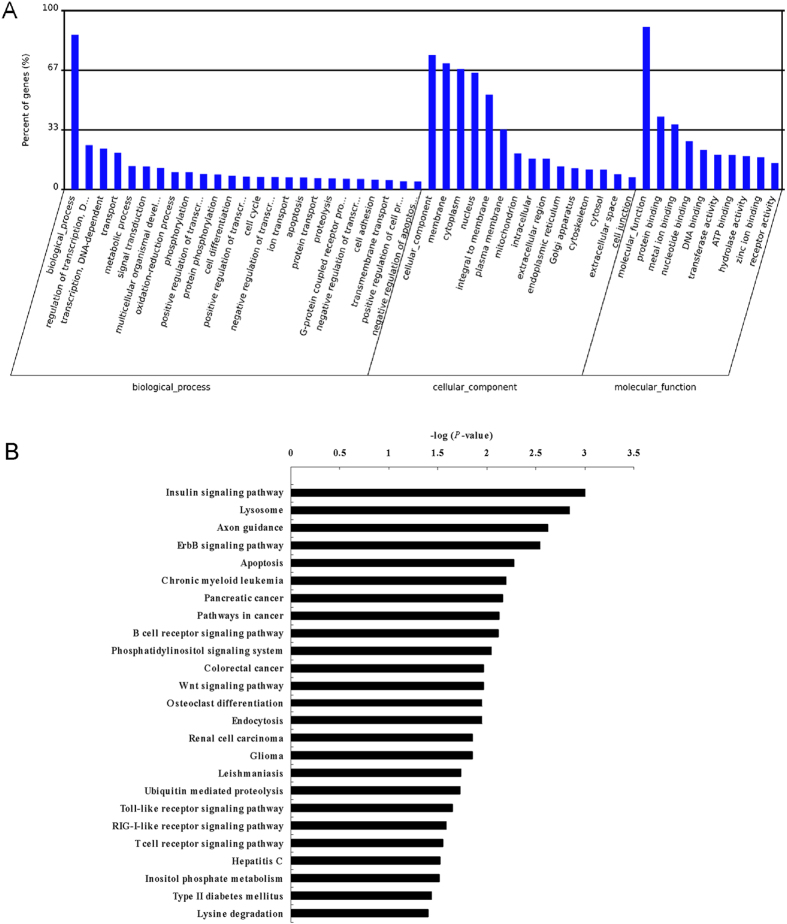
Bioinformatic analysis of predicted targets of all differentially regulated miRNAs. (**A**) GO category analysis based on predicted targets of all differentially regulated miRNAs. The vertical axis represents the GO category, and the horizontal axis represents the enrichment of GO. (**B**) Pathway analysis for the differentially regulated miRNAs. Only pathways with *P* < 0.05 are shown. The vertical axis represents the pathway category, and the horizontal axis represents the value of -Log*P*.

We performed a gene ontology (GO) term and Kyoto Encyclopedia of Genes and Genomes (KEGG) pathway annotation of the predicted miRNA targets using gene annotation tool. The high-enrichment GO terms were regulation of transcription, transferase activity, protein phosphorylation, negative regulation of canonical Wnt receptor signaling pathway, Wnt receptor signaling pathway, etc (Fig. 2A). KEGG pathway analysis identified 25 pathways that were over-represented (*P* < 0.05) (Fig. 2B), including insulin signaling pathway, apoptosis, Wnt signaling pathway, etc. Thus, the GO term and KEGG pathway annotations for the predicted miRNA targets illustrated the likely roles for these differentially expressed miRNAs during nonalcoholic fibrosing steatohepatitis.

### Validation of miRNA expression by quantitattive real-time PCR (qRT-PCR)

In accordance with the microarray assay results, qRT-PCR confirmed that miR-15b-5p was significantly up-regulated, whereas miR-146a-5p and miR-203-3p were down-regulated in fibrosing steatohepatitis. Given miR-146a-5p was one of the most significant down-regulated miRNAs in fibrosing steatohepatitis, we chose it as a candidate for further investigation (Fig. 1D).

### miR-146a-5p was down-regulated in the activated HSCs *in vitro*

**Figure 3:**
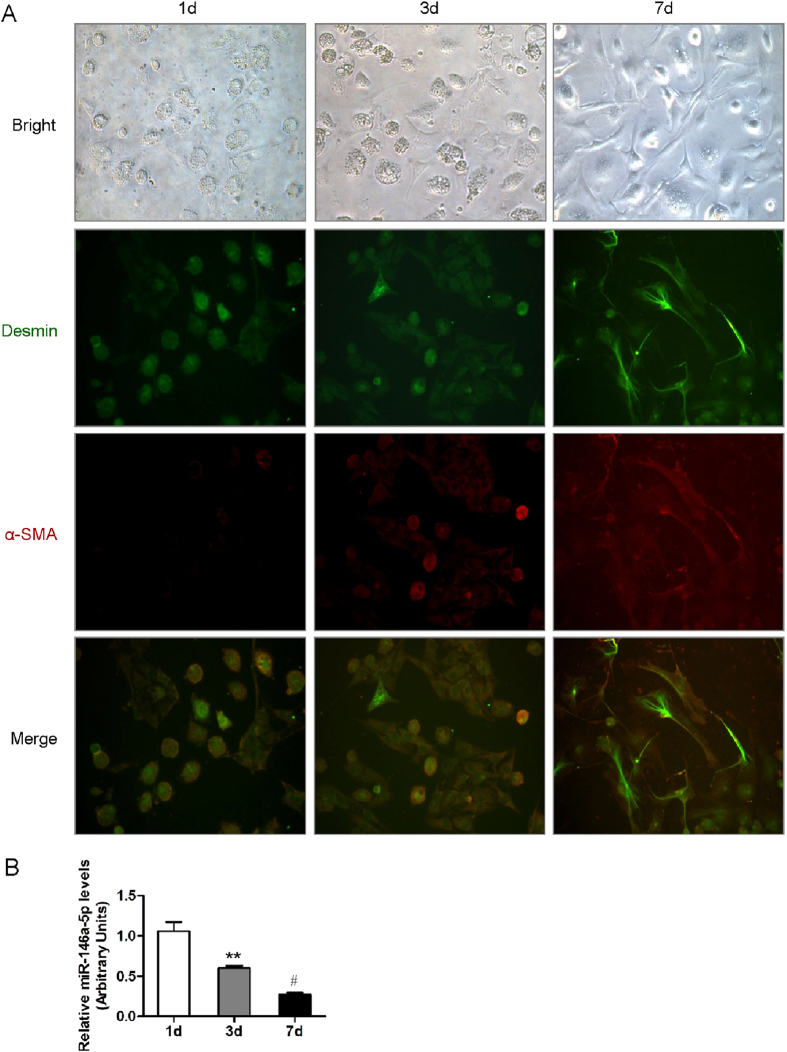
miR-146a-5p was downregulated in activated hepatic stellate cells (HSCs). (**A**) Representative morphological images and Immunocytochemistry staining of quiescent and activated HSCs for desmin and α-SMA (400×). (**B**) miR-146a-5p expression was examined by real-time qRT-PCR. Values are mean ± SD, **P < 0.01 compared with 1d, ^#^*P* < 0.05 compared with 3d.

We further isolated primary HSCs in mice liver. On average, 5 × 10^6^ HSCs were harvested from each mouse. The purity of freshly isolated living HSCs was more than 95% determined by the trypan blue exclusion method. The immunofluorescent staining of α-SMA, a marker of HSC activation, showed a significant increase in primary activated HSCs *in vitro* (Fig. 3A). The expression of miR-146a-5p was found to be significantly down-regulated in the activated HSCs (Fig. 3B).

### Overexpression of miR-146a-5p suppressed proliferation of HSCs

**Figure 4:**
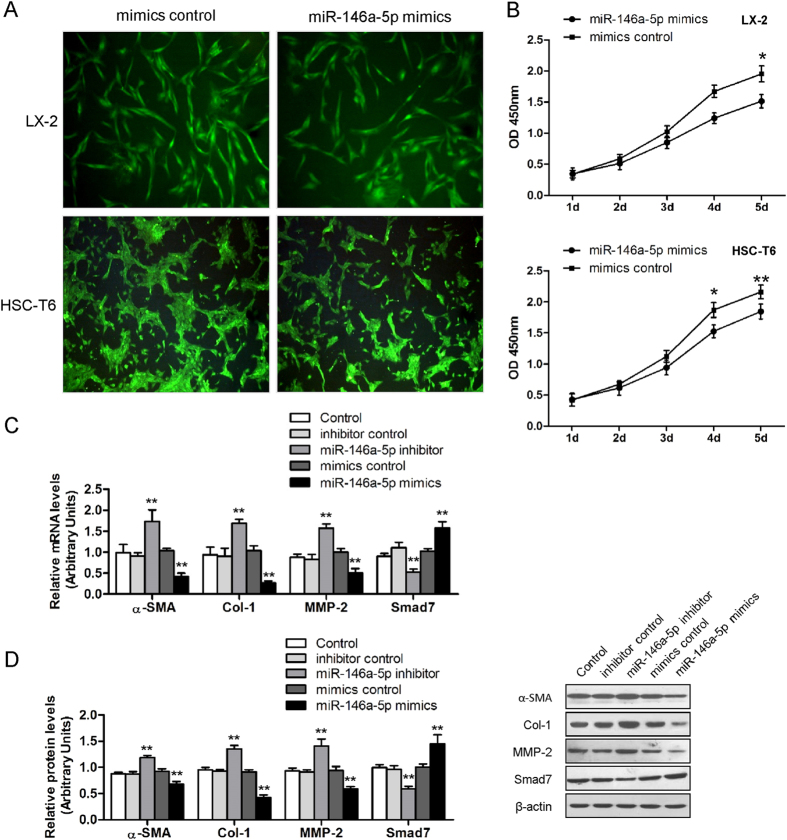
miR-146a-5p inhibited cell proliferation and cell growth. (**A**) Desmin staining indicating LX-2 and HSC-T6 cell proliferation decrease when miR-146a-5p was overexpressed. (**B**) miR-146a-5p inhibited LX-2 and HSC-T6 cell growth as determined by CCK-8 assays. Values are mean ± SD, *P < 0.05, **P < 0.01 compared with mimics control. (**C**) Effect of miR-146a-5p on mRNA and (**D**) protein expression of fibrogenic genes α-SMA, Col-1, MMP-2 and Smad7. β-actin was used as loading control. Values are mean ± SD, **P < 0.01 compared with control.

In light of the decreased expression of miR-146a-5p in activated HSCs, we next investigated the effect of miR-146a-5p on the proliferation of two HSC cell lines LX-2 and HSC-T6. The miR-146a-5p expression was markedly up-regulated by miR-146a-5p mimics (Supplementary Figure S1). As determined by desmin immunofluorescence and CCK-8 assay, overexpression of miR-146a-5p led to an inhibition of cell proliferation in LX-2 and HSC-T6 as compared to the control cells (Fig. 4A,B).

### Effects of miR-146a-4p on HSC activation and collagen deposition

To clarify the roles of miR-146a-5p overexpression in HSC activation and collagen deposition, we transfected miR-146a-5p mimics into HSCs. As shown in Fig. 4C,D, overexpression of miR-146a-5p significantly suppressed mRNA and protein expression of α-SMA, Col-1, matrix metalloproteinase-2 (MMP-2) and up-regulated the mRNA and protein expressions of Smad7, which were characterized genes in the activation process of HSCs. On the contrary, the mRNA and protein expressions of these genes were reversed by miR-146a-5p silence (Fig. 4C,D) suggesting that miR-146a-5p may suppress HSC activation as well as ECM deposition.

### miR-146a-5p directly acted on the 3’UTR of Wnt1 and Wnt5a mRNA

**Figure 5:**
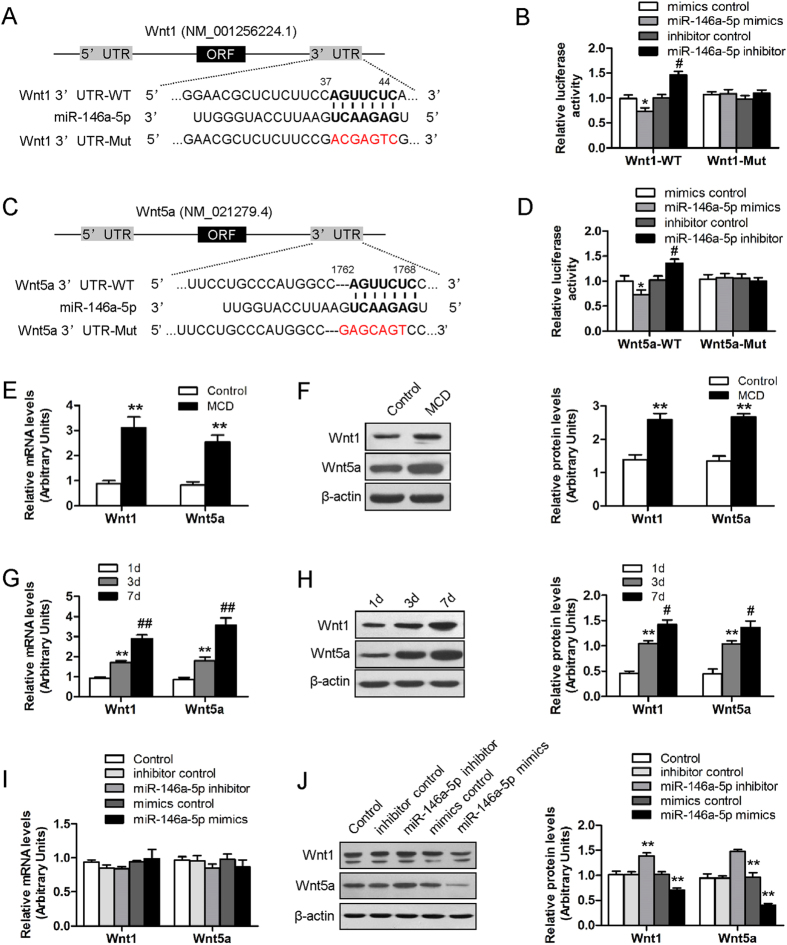
Experimental validation of Wnt1 and Wnt5a as target genes of miR-146a-5p. (**A**,**C**) miR-146a-5p potential binding sites on the 3’UTR of Wnt1 and Wnt5a. (**B**,**D**) HEK-293T cells were transfected with luciferase reporter vector containing either wild-type or mutant form of 3’UTR of Wnt1 and Wnt5a, in the presence of either miR-146a-5p mimics, mimics control, miR-146a-5p inhibitor and inhibitor control, and then assessed for luciferase reporter activity at 48 hours post-transfection. Values are mean ± SD, *P < 0.05 compared with mimics control, ^#^*P* < 0.05 compared with inhibitor control. (**E**) Hepatic mRNA and (**F**) protein expressions of Wnt1 and Wnt5a were up-regulated in MCD-fed mice as compared to the control. (**G**) mRNA and (**H**) protein expression of Wnt1 and Wnt5a were increased in the process of HSC activation. β-actin was used as loading control. Values are mean ± SD, **P < 0.01 compared with 1d, ^#^*P* < 0.05 compared with 3d, ^##^*P* < 0.01 compared with 3d. (**I**) HSC-T6 cells were transfected with miR-146a-5p inhibitor or inhibitor control, miR-146a-5p mimics or mimics control for 48 h. mRNA and (**J**) protein expression of Wnt1 and Wnt5a were reduced by miR-146a-5p mimics and increased by miR-146a-5p inhibitor in HSC-T6 cells. β-actin was used as loading control. Values are mean ± SD, **P < 0.01 compared with control.

Among the predicted targets of miR-146a-5p, Wnt1 and Wnt5a were predicted as potential target genes of miR-146a-5p. Examination of the homology showed that the 7 nucleotides in the seed region of miR-146a-5p were complementary to bases of Wnt1 (Fig. 5A) and Wnt5a (Fig. 5C), respectively. No perfect binding site predicted for miR-146a-5p in the coding region of Wnt1 and Wnt5a was identified. To determine whether miR-146a-5p directly binds to the predicated sites of the Wnt1 and Wnt5a 3′UTR, we performed luciferase reporter assays. miR-146a-5p mimics significantly reduced Wnt1 3′UTR-dependent and Wnt5a 3′UTR-dependent luciferase activity but not affect mutant reporter luciferase activity, whereas mimic control had no effect on wild type or mutant reporter luciferase activity. On the other hand, miR-146a-5p inhibitor enhanced wild type or mutant reporter luciferase activity (Fig. 5B,D). These results suggested the interaction between miR-146a-5p and the 3’UTR of Wnt1 and Wnt5a mRNA.

### miR-146a-5p down-regulated Wnt1 and Wnt5a at the posttranscriptional level

We further explored the regulation level of miR-146a-5p on Wnt1 and Wnt5a gene expression. Accompanied with the down-regulation of hepatic miR-146a-5p, the mRNA and protein expressions of Wnt1 and Wnt5a were markedly elevated in mice fed with MCD diet compared with mice fed with control diet (Fig. 5E,F). The consistent results were observed *in vitro*. MiR-146a-5p target genes Wnt1 and Wnt5a were significantly enriched in HSC activation (Fig. 5G,H). Wnt1 and Wnt5a protein but not mRNA expression was significantly decreased in miR-146a-5p mimic-transfected-HSCs compared with the mimic control-transfected-HSCs. Silencing miR-146a-5p by miR-146a-5p inhibitor increased the expression of Wnt1 and Wnt5a protein levels (Fig. 5I,J). These results suggest that miR-146a-5p regulated Wnt1 and Wnt5a genes expression at the posttranscriptional level.

### Effects of overexpression and inhibition of miR-146a-5p on the downstream target genes of Wnt signaling pathway

**Figure 6:**
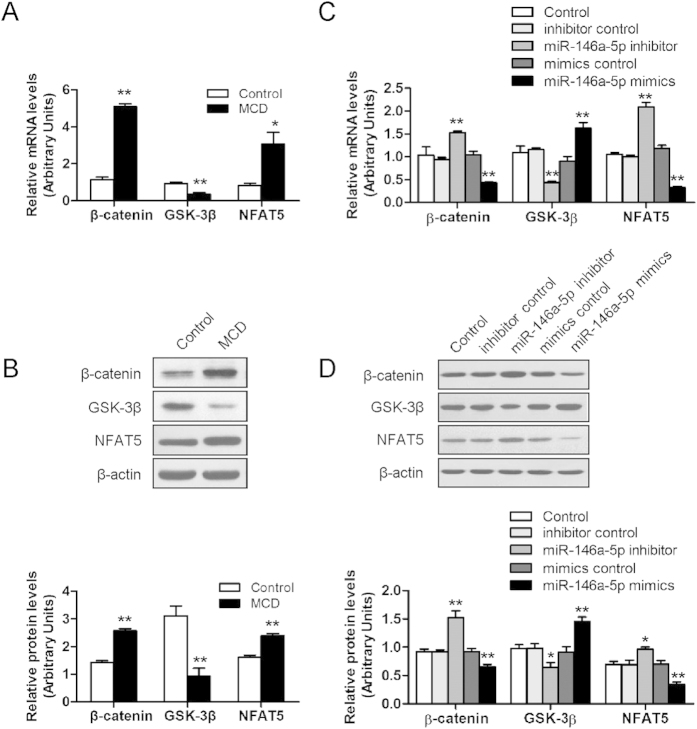
miR-146a-5p regulates the downstream genes of Wnt signaling pathway. (**A**) mRNA and protein (**B**) levels of β-catenin, GSK-3β and NFAT5 in mice liver fed MCD diet and the controls. (**C**) HSC-T6 were transfected with miR-146a-5p inhibitor or inhibitor control, miR-146a-5p mimics or mimics control for 48 h. mRNA and protein (**D**) levels of β-catenin, GSK-3β and NFAT5 were analysis by real-time RT-PCR and western blot, respectively. β-actin was used as loading control. Values are mean ± SD, **P < 0.01 compared with control.

To investigate whether down-regulation of Wnt1 and Wnt5a by miR-146a-5p could affect the downstream factors in Wnt signaling pathway, we analyzed the β-catenin, glycogen synthase kinase-3β (GSK-3β), and nuclear factor of activated T-cells 5 (NFAT5) mRNA and protein levels by qRT-PCR and Western blot, respectively. As compared to the control, the hepatic mRNA and protein expressions of β-catenin and NFAT5 were significantly increased, GSK-3β expressions was significantly decreased (Fig. 6A,B). *In vitro*, miR-146a-5p mimics significantly down-regulated β-catenin and NFAT5 expressions, and upregulated GSK-3β expression on both mRNA and protein levels, and silencing of miR-146a-5p showed the contrary changes (Fig. 6C,D), indicating that miR-146a-5p might modulate Wnt signaling pathway.

### Knockdown of Wnt1 or Wnt5a inhibited the gene expressions of Wnt signaling downstream and fibrogenesis

**Figure 7:**
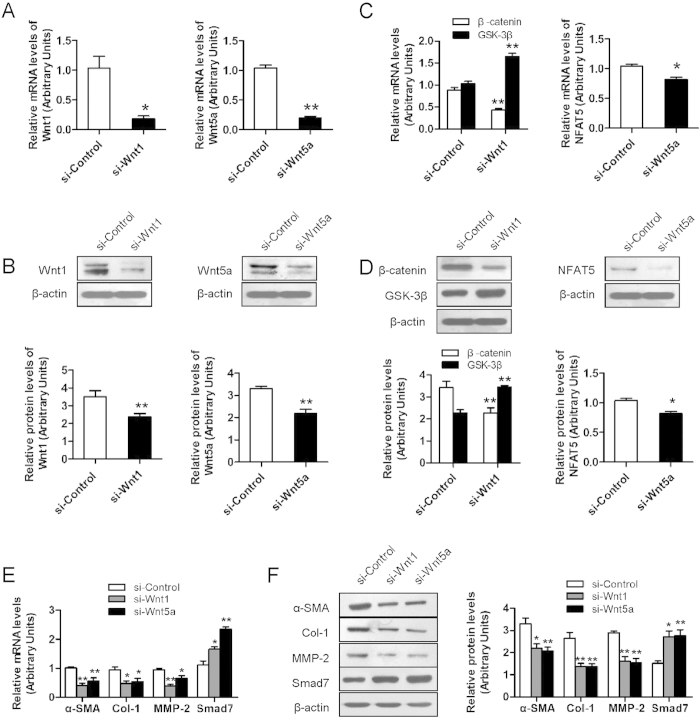
Knockdown of Wnt1 or Wnt5a inhibits Wnt signaling pathway and reduces HSC activation. (**A**) Knockdown efficiency of Wnt1 or Wnt5a in HSC-T6 cells was confirmed by RT-PCR and (**B**) Western blot. (**C**) Knockdown of Wnt1 or Wnt5a inhibits the mRNA and protein expressions (**D**) of β-catenin, NFAT5, and increase the expression of GSK-3β. (**E**) Knockdown of Wnt1 or Wnt5a inhibits the mRNA and protein levels (F) of α-SMA, Col-1 and MMP-2, and enhanced the expression of Smad7. β-actin was used as loading control. Values are mean ± SD, *P < 0.05, **P < 0.01 compared with control.

To seek an explanation for the effect of Wnt1 or Wnt5a on liver fibrosis, we knockdown Wnt1 and Wnt5a in HSC-T6 cells by siRNA transfection. QRT-PCR and Western blot confirmed that Wnt1 and Wnt5a were significantly downregulated after siRNA transfection (Fig. 7A,B). Knockdown of Wnt1 or Wnt5a significantly reduced the mRNA and protein expressions of β-catenin and NFAT5, and elevated GSK-3β expression (Fig. 7C,D). Moreover, it was also observed that knockdown of Wnt1 or Wnt5a significantly suppressed pro-fibrotic genes α-SMA, Col-1 and MMP-2 expressions and enhanced an inhibitory Smad, Smad7 expression (Fig. 7E,F).

## Discussion

The MCD model is a well-known rodent model for the study of steatohepatitis and liver fibrosis. The advantage of MCD diet model was being more efficient and reproducible for inducing severe liver damage and progressive fibrosis. Following MCD diet for 8 weeks, mice rapidly and consistently developed a severe pattern of steatohepatitis with mixed inflammatory cell infiltration, fibrosis in the pericellular, perisinusoial and portal area. Thus, this diet approach models the subgroup of NASH patients with histologically advanced NASH, and it is ideal for studying mechanisms driving NASH-related inflammation/fibrosis.

In this study, we identified the aberrant expression of miRNAs in nonalcoholic fibrosing steatohepatits in mice. Among the differential expressed microRNAs, we identified 19 up-regulated and 18 down-regulated miRNAs (>2-fold changes) in fibrosing steatohepatits. Coordination of aberrant expression of these miRNAs may contribute to hepatic fibrosis in nonalcoholic steatohepatits. The potential targets of differentially expressed miRNAs were known to play a role in the regulation of signal transduction, cell proliferation, differentiation and apoptosis. The KEGG signaling pathway analysis of the miRNA microarray data was performed to cluster the signaling information involving predicted targets of the differential miRNAs. Oxidative stress, cell apoptosis, Wnt signaling and Toll-like receptor pathway are enriched in MCD diet induced hepatic fibrosis. All these genes and signaling pathways may play key roles in developmental processes of nonalcoholic fibrotic steatohepatits.

It was proved consistent by both the microarray assay and the real-time PCR that the expression of miR-146a-5p and miR-203-3p were significantly down-regulated and miR-15b-5p expression was significantly up-regulated in the fibrotic liver in mice. The effects of various miRNAs on liver fibrosis have been illustrated in recent studies. miR-29 was reported to play a crucial role in CCL4-induced liver fibrosis^18^. miR-199 and 200 family was up-regulated in the progression of liver fibrosis^19^. Moreover, miRNAs were demonstrated to play an important role in regulating HSCs function, such as activation, cell proliferation and production of ECM. miR-27a/b maintain HSCs a more quiescent phenotype and inhibit cell proliferation^20^. Overexpression of miR-122 in HSCs led to significant inhibition of the production of mature Col-1^21^. In our study, among the validated miRNAs, hepatic miR-146a-5p showed the most significantly dysregulated. Similarly, the down-regulation of miR-146a-5p was observed in the activation process of primary HSCs isolated from the mouse livers compared to their quiescent phenotype. Collectively, these data suggested that miR-146-5p might play a crucial role in the development of nonalcoholic fibrosing steatohepatitis and its suppression may be associated with HSC activation.

As miR-146a-5p expression was down-regulated during the HSC activation and hepatic fibrogenesis, the effect of miR-146a-5p on the biological behavior of HSCs was evaluated. We transducted miR-146a-5p mimics into activated human (LX-2) and rat (T6) HSCs. Notably, the cell proliferation assay by CCK-8 results revealed that miR-146a-5p significantly inhibited the proliferation of both HSC cell lines, which could be also confirmed by desmin immunofluorescent staining. In fact, activation of HSCs disrupts the balance between matrix production and degradation^2^. In our study, miR-146a-5p inhibited pro-fibrotic genes α-SMA, Col-1 and MMP-2 expressions, enhanced the expression of Smad7, a negative mediators of TGF-β signaling. MMPs, a family of ECM degradative enzymes, are promptly expressed by activated HSCs^22^. MMP-2 promotes hepatic fibrosis by increasing HSC proliferation and it is required for degradation of the accumulated collagen. Smad7 is a crucial negative regulator of TGF-β signaling, and antagonizes the activity of the Smad4. Increased Smad7 expression could attenuate the fibrogenic response of hepatic stellate cells induced by TGF-β1^23^. In line with our results, another investigation revealed a potential mechanism for the role of miR-146a in fibrosis through regulating the expression of Smad4^24^. These findings implied that miR-146a-5p might negatively regulated liver fibrogenesis through inhibiting the HSC activation and collagen synthesis in HSCs.

In consideration of the important role of miR-146a-5p in repressing liver fibrogenesis through suppressing the activation of HSCs, we investigated the impact of Wnt1, Wnt5 and thier downstream effectors participating in this function. In fact, one single miRNA can regulate tens to hundreds targeted genes. Wnt1 and Wnt5a were predicted to be direct targets of miR-146a-5p by bioinformatics analysis and were confirmed with dual luciferase report assay and western blot. Moreover, miR-146a-5p overexpression in HSCs resulted in up-regulation of Wnt1 and Wnt5a in protein levels but no change in mRNA expression, confirming that miR-146a-5p regulated Wnt1 and Wnt5a expression at a post-transcription level. Wnt1 and Wnt5a are key elements of the canonical and non-canonical Wnt signaling pathway, respectively^25^. Wnt signaling promotes hepatic fibrosis by enhancing activation and survival of HSCs and is one of the major signal transduction pathways associated with hepatic fibrogenesis^26^,^27^,^28^. In our study, knockdown of Wnt1 or Wnt5a led to the suppression of α-SMA, Col-1, MMP-2 and increase of Smad7 expression, which was consistent with the efficacy of miR-146a-5p. To demonstrate whether Wnt1 and Wnt5a are major targets to mediate the activity of miR-146a-5p, we used a combined loss-of-function approach to functionally characterize Wnt1 and Wnt5a in fibrogenesis. Results showed that knockdown of Wnt1 or Wnt5a mimicked the roles of miR-146a-5p, further indicating that Wnt1 and Wnt5a are the primary functional targets of miR-146a-5p in nonalcoholic fibrosing steatohpatitis.

Given that Wnt signaling was reported to maintain the quiescent state of HSCs^29^ and Wnt1 and Wnt5a were demonstrated to be the targets of miR-146a-5p for suppressing the activation of HSCs, we further investigated the possible downstream effectors participating in this function. The studies have shown that Wnt1 and Wnt5a exerted their effects by regulating β-catenin, GSK-3β and NFAT5, respectively. Our data indicated that miR-146a-5p inhibited β-catenin, NFAT5 and activate GSK-3β by down-regulation of Wnt1 and Wnt5a. β-catenin, a core component of canonical Wnt signaling, has been shown to be an important regulator of cellular proliferation and differentiation^30^. The knockdown of β-catenin in HSC-T6 cells inhibited cells proliferation and synthesis of Col-1 and Col-3^31^. This might be another reason for the inhibition effect of miR-146a-5p on HSC proliferation and collagen secretion. GSK-3β, a negative regulator of canonical Wnt signaling^32^, is implicated in various biological processes including cell growth, differentiation and apoptosis^33^. Inhibition of GSK-3β impeded synthesis of α-SMA by reducing β-catenin phosphorylation in HSCs^29^. NFAT5 is a transcriptional target of Wnt5a signaling, was reported to accelerate cell proliferation^34^ and could also stimulate the production of TGF-β1 and subsequent activation of Smad3^35^. In the present study, knockdown of Wnt1 or Wnt5a inhibited the expression of downstream factors, β-catenin, NFAT5, and increased the expression of GSK-3β. Taken together, the results implicated a possible mechanism that miR-146a-5p negatively modulated Wnt signaling pathway via direct interaction with Wnt1 and Wnt5a.

In conclusion, we identified hepatic miRNAs and evaluated their expression patterns in nonalcoholic fibrosing steatohepatitis induced by MCD diet using microarray. Among the validated miRNAs, miR-146a-5p was significant down-regulated in nonalcoholic fibrosing steatohepatitis and in activated HSCs. Overexpression of miR-146a-5p contributed to the development of liver fibrosis through inhibiting the proliferation, activation of HSCs and deposition of collagen, suppressing Wnt signaling pathway. Therefore, miR-146a-5p might serve as a novel regulator in the pathogenesis of nonalcoholic fibrosing steatohepatitis.

## Methods

### Animal models of nonalcoholic fibrosing steatohepatitis

Eight-week-old male C57BL/6J mice were bred and housed as previously described. Nonalcoholic fibrosing steatohepatitis was induced by feeding the mice with MCD diet (Research diets, Inc., NJ, New Brunswick, USA) for 8 weeks. Meanwhile, mice were fed with diet supplemented with choline bitartate and DL-methionine (Research diets, Inc., NJ, New Brunswick, USA) as controls. At the end of the experiment, all the animals were sacrificed under anesthesia, and blood samples were collected from femoral artery for biochemical analysis. The livers were partly fixed in 10% formalin for histological analysis or snap-frozen in lipid nitrogen followed by storage at −80 °C freezer until required. All the protocols and procedures were performed following the guidelines of Hebei Committee for Care and Use of Laboratory Animals and were approved by the Animal Experimentation Ethics Committee of Hebei Medical University.

### Histological analysis and Biochemical analysis

Hematoxylin and eosin stained and Masson trichromatism stained paraffin-embedded liver sections (5 μm thick) were scored for hepatic steatosis, inflammation and fibrosis as described previously in accordance with the Brunt’s criteria and the histological scoring system for NAFLD issued by the Pathology Committee of the Nonalcoholic Steatohepatitis Clinical Research Network. Serum ALT and AST levels were measured by the enzymatic kinetic method using an automatic biochemical analyzer (Olympus AU2700, Japan) according to the manufacturer’s instructions.

### MicroRNA microarray Assay

Total RNA was extracted from 20 mg liver tissue of MCD diet-fed mice and control diet-fed mice (n = 3 mice/group) using TRIzol reagent (Invitrogen) according to the manufacturer’s instructions. The μParaflo™ MicroRNA microarray Assay was performed using a service provider (LC Sciences, Houston, TX, USA). The assay started from 4 to 8 μg total RNA sample were 3’-extended with a poly (A) tail using poly (A) polymerase. An oligonucleotide tag was then ligated to the poly (A) tail for later fluorescent dye staining. Hybridization was performed overnight on a μParaflo microfluidic chip using a micro-circulation pump (Atactic Technologies). After RNA hybridization, tag-conjugating Cy3 dye was circulated through the microfluidic chip for dye staining. Fluorescence images were collected using a laser scanner (GenePix 4000B, Molecular Device) and digitized using Array-Pro image analysis software (Media Cybernetics). Data was analyzed by first subtracting the background and then normalizing the signals using a LOWESS filter (Locally-weighted Regression).

### QRT-PCR analysis

Total RNA was isolated and extracted from frozen liver tissues with TRIzol reagent (Invitrogen). cDNA was synthesized using reverse transcriptase with miRNAs-specific stem-loop primer (RiboBio, Guangzhou, China) or oligo dT primers (Thermo, Waltham, MA, USA). Differentially qRT-PCR was performed on an ABI 7500 Real-Time PCR system (Applied Biosystems, Foster City, CA, USA) using SYBR Green master mixture (CoWin Biotech, Beijing, China). The relative abundance of miRNA was normalized to small nuclear RNA U6, and the expression levels of genes were normalized against an endogenous reference gene glyceraldehyde-phosphate dehydrogenase (GAPDH). The relative amount of each miRNA and genes were measured using the 2^−ΔΔCt^ method. All qRT-PCR reactions were conducted in triplicate. The primers used for qRT-PCR are shown in Table 1.

### Identification of potential miRNA gene targets

Predicted gene targets of all differentially expressed miRNAs were identified using three databases including TargetScan, PicTar, and miRanda^36^. All predicted targets indentified in any database were then subjected to gene GO (www.geneontology.org) analysis to uncover the miRNA-gene regulatory network on the basis of biological processes and molecular functions^37^. Enrichment provided a measure of the significance of the function. Pathway analysis was used to determine the significant pathways of the differential genes according to KEGG. Fisher’s exact test and χ^2^ test were used to classify the GO category and the significant pathway. The false discovery rate (FDR) was calculated to correct the *P*-value. *P*-value of <0.001 and a FDR of <0.05 was considered to statistically difference.

### Isolation, culture, and identification of HSCs

Primary HSCs were isolated from 12- to 16-week-old male C57BL/6J mice (weighted about 30 g) *in situ* perfusion with pronase-collagenase digestion and fractionation on a discontinuous gradient of Percoll (50% and 25%). Stellate cells were harvested from the 25% media interface. Cell purity and viability were confirmed by trypan blue staining. HSCs were cultured in Dulbecco’s modified Eagle medium (DMEM, GIBCO, Grand Island, NY, USA) supplemented with 10% fetal bovine serum (FBS, BioInd, Israel), 100 U/L penicillin, 100 μg/mL streptomycin, and 2 mmol/L glutamine in a humidified 5% CO_2_ atmosphere. On average, 5 × 10^6^ HSCs were harvested from each mouse. The purity of freshly isolated living HSCs was more than 95% determined by the trypan blue exclusion method.

### miR-146a-5p transfection in HSCs

The HSCs (LX-2, HSC-T6) cells were seeded at a density of 2 × 10^5^/ml and the medium was replaced with fresh DMEM medium without antibiotics. 50 nM miR-146a-5p mimic or 100 nM miR-146a-5p inhibitor (Ribo Bio, Guangzhou, China) was transfected into HSCs using lipofectamine 2000 (Invitrogen, Carlsbad, CA, USA) for gain and loss of miR-146a-5p function experiments, respectively. The corresponding negative sequence (mimic control or inhibitor control) were used with the same concentration as controls in miRNA experiments. After 5 h culture with transfection mix, the cell culture medium was replaced with 10% FBS/DMEM with antibiotics. At 48 h after the transfection, cells were harvested by mild trypsinization, washed in phosphate-buffered saline. All experiments were repeated in triplicate.

### RNA interference and transfection

HSC-T6 cells were transfected with siRNA against Wnt1 or siRNA against Wnt5a, and control siRNA (Ribo Bio, Guangzhou, China) consisting of a scrambled sequence that will not lead to specific degradation of any cellular message. The siRNAs were transfected into HSC-T6 cells using lipofectamine 2000 (Invitrogen). Knockdown efficiency was evaluated by qRT-PCR and Western blot. The synthesized oligos were shown in Table 1

### Immunocytochemistry analysis

HSCs were grown on chamber slides, and the transfection experiments were carried out as described before. The cells were fixed in 4% paraformaldehyde for 15 min and washed with PBS three times. The cells were permeated with X-Triton100 for 20 min and washed with PBS three times. Then the cells were blocked with 5% bovine serum albumin in PBS for 1 h followed by incubation with primary antibodies against desmin (ProteinTech Group, Chicago, USA) and α-SMA (Novus Biologicals, Littleton, USA) for 16 h at 4 °C overnight. After washing with PBS three times, secondary antibody was applied and incubated for 1 h. After additional washing, the cells were analyzed by fluorescence microscopy.

### Cell proliferation assay

Five hours after transfection with miR-146a-5p mimics or mimics control, LX-2 cells and HSC-T6 were reseeded on 96-well plates, at a density of 5 × 10^3^ cells per well for 1, 2, 3, 4, 5d. The cells were assayed for proliferation using the Cell Counting Kit-8 (CCK-8, Dojindo, Kumamoto, Japan), according to the manufacturer’s instructions. The experiments were conducted three times independently.

### Western blot analysis

Total proteins were extracted from liver tissue and cells using radio-immunoprecipitation buffer (RIPA). 80 μg of sample proteins were separated by in 10 or 12% SDS-PAGE gel, and transferred onto PVDF membranes (Millipore Corporation, Billerica, MA, USA) by electrobloting. The membranes were blocked for 60 min in a buffer containing 0.1% Tween-20 and 5% milk. The membranes were incubated overnight at 4 °C with primary antibodies against α-SMA, Col-1, MMP-2 (Bioss, Beijing, China), Smad7 (Novus Biologicals, Littleton, USA), Wnt1 (Abcam, Cambridge, MA, USA), Wnt5a (Novus Biologicals, Littleton, USA), β-catenin, GSK-3β (ProteinTech Group, Chicago, USA), NFAT5 (Santa Cruz, CA, USA). Immune complexes were detected with horseradish peroxidase (HRP)-conjugated secondary antibodies (ProteinTech Group, Chicago, USA), and then were visualized by ECL method. β-actin (Boster, Wuhan, China) was served as a loading control. Intensity of each protein band of interest was quantified by densitometry using Quantity One 4.6.3 software (Bio Rad).

### Luciferase activity assay

Sequence of segments with the wild-type (WT) or mutant (Mut) seed region of Wnt1 and Wnt5a were synthesized and cloned into psiCHECK^TM^-2 luciferase vector (Promega, Madison, WI, USA) between Xho I and Not I restriction sites. An empty luciferase reporter vector was used as a negative control. HEK-293T cells were cultured in 24-well plated and each well was transfected with 200 ng of the respective psi-CHECK2 3′UTR constructs, and 50 nM miR-146a-5p mimics or mimics control, and 100 nM inhibitor or inhibitor control, using lipofectamine 2000 transfection reagent (Invitrogen, Carlsbad, CA), according to manufacturer’s protocol. After 5 hours, OptiMEM (Invitrogen, CA, USA) transfection medium was replaced with DMEM. Cells were harvested and assayed 48 hours after transfection using the Luciferase Assay System (Promega). The synthesized oligos were shown in Table 1.

### Statistical analysis

The data are expressed as the mean ± standard deviation. Statitistical analysis was performed using SPSS 17.0. One-way analysis of variance (ANOVA) test and Student’s test were used for statistical analysis. *P* < 0.05 was considered to indicate a statistically significant difference.

## Additional Information

**How to cite this article**: Du, J. *et al.* MiR-146a-5p suppresses activation and proliferation of hepatic stellate cells in nonalcoholic fibrosing steatohepatitis through directly targeting Wnt1 and Wnt5a. *Sci. Rep.* **5**, 16163; doi: 10.1038/srep16163 (2015).

## Supplementary Material

Supplementary Information

## Figures and Tables

**Table 1:** Primers for real-time quantitative PCR analysis.

Gene	Length of production (bp)	Primers
Wnt1	116	F 5′- CTGTGCGAGAGTGCAAATGG -3′
		R 5′- GATGAACGCTGTTTCTCGGC -3′
Wnt1-3′UTR	919	F 5′- CCGCTCGAGCCCTCACGACCTCGTCTACT -3′
		R 5′- ATTTGCGGCCGCGGCCACATTTCGGTGCAAAT -3′
Wnt1-3′UTR mut	919	F 5′- GCTCTCTTCCGACGAGTCGACACACTCGCTGGTC -3′
		R 5′- CGAGTGTGTCCTGAGCAGGAAGAGAGCGTTCCCG -3′
Wnt5a	621	F 5′- GTGATGCAAATAGGCAGCCG -3′
		R 5′- GTCCATCCCCTCTGAGGTCT -3′
Wnt5a-3′UTR	472	F 5′- CCGCTCGAGACAGTTTTCTCTGGGCTTGC -3′
		R 5′- ATTTGCGGCCGCCACTTTTGAAACAGAGGTGCCC -3′
Wnt5a-3′UTR mut	472	F 5′- GCCCATGGCCGAGCAGTCACCCTCTCTTTGGTGT-3′
		R 5′- AGAGAGGGTGTGACGAGGGCCATGGGCAGGAAGC-3′
β-catenin	137	F 5′- CAGATCCCATCCACGCAGTT -3′
		R 5′- ATTGCACGTGTGGCAAGTTC -3′
GSK-3β	158	F 5′- CGAACTCCACCAGAGGCAAT -3′
		R 5′- AAGAGTGCAGGTGTGTCTCG -3′
NAFT5	178	F 5′- TCTCACACATCCAGACCCCT -3′
		R 5′- TGTACAAAGGCTCTGTCGCT -3′
α-SMA	98	F 5′- CTGACAGAGGCACCACTGAA-3′
		R 5′- CATCTCCAGAGTCCAGCACA-3′
Col-1	308	F 5′- GGGCGAGTGCTGTGCTTT -3′
		R 5′- GACCCATTGGACCTGAACC -3′
MMP-2	103	F 5′- TTTCTATGGCTGCCCCAAGG -3′
		R 5′- GTCAAGGTCACCTGTCTGGG -3′
Smad7	153	F 5′- TTTAAACCCGGGCTCCATCC -3′
		R 5′- GGAGGGGACCGAGTAGACTT -3′
GAPDH	233	F 5′- GGTGAAGGTCGGTGTGAACG -3′
		R 5′- CTCGCTCCTGGAAGATGGTG -3′
