# Supplementary material for: MiR-146a-5p suppresses activation and proliferation of hepatic stellate cells in nonalcoholic fibrosing steatohepatitis through directly targeting Wnt1 and Wnt5a
Source: Sci Rep. 2015 Nov 5;5:16163. doi: 10.1038/srep16163 (PMC4633641; doi:10.1038/srep16163)
Supplement: Supplementary Information [file srep16163-s1.pdf]

MiR-146a-5p suppresses activation and proliferation of hepatic stellate cells in nonalcoholic fibrosing steatohepatitis through directly targeting Wnt1 and Wnt5a

Jinghua Du, Xuemin Niu, Yang Wang, Lingbo Kong, Rongqi Wang, Yuguo Zhang, Suxian Zhao, Yuemin Nan*

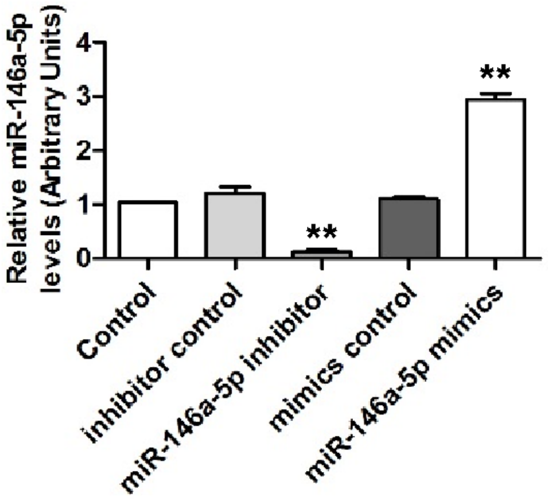

Supplementary Figure 1: The miR-146a-5p expression was regulated by miR-146a-5p inhibitor and mimics. The expression of miR-146a-5p was validated by qRT-PCR after transfected with miR-146a-5p inhibitor and mimics. The miR-146a-5p expression was significantly down-regulated by miR-146a-5p inhibitor and was up-regulated by miR-146a-5p mimics. Values are mean ± SD, ***P* < 0.01 compared with control.
